# Trends in referrals to liaison psychiatry teams from UK emergency departments for patients over 65

**DOI:** 10.1192/bjo.2021.823

**Published:** 2021-06-18

**Authors:** Sarah Bradbury, George Crowther, Manimegalai Chinnasamy, Laura Shaw, Sara Ormerod, Alison Wilkinson, Rebecca Chubb, Mazen Daher, Pramod Kumar, Andrew Gaskin, Karen Williams, Angus Brown, Eleanor Stebbings, Sunita Sahu, Roger Smyth, Hilary Kinsler, Stephen O'Connor, Andrew Wells, Ross Overshott, Kehinde Junaid, Aparna Mordekar, Jenny Humphries, Karen James, Shweta Mittal, Sarita Dasari, Hugh Grant-Peterkin, Niall Campbell, Robert West, Professor George Tadros, Elizabeth Sampson

**Affiliations:** 1 Humber Teaching NHS Foundation Trust; 2 Leeds and York Partnership NHS Foundation Trust, University of Leeds; 3 Bradford District Care Trust; 4 Tees, Esk and Wear Valleys Foundation NHS Trust; 5 Birmingham and Solihull Mental Health NHS Foundation Trust; 6 Cambridgeshire and Peterborough NHS foundation trust; 7 North Staffordshire Combined Healthcare Trust; 8 East London NHS Foundation Trust; 9 Berkshire Healthcare NHS Foundation Trust; 10 South London and Maudsley NHS Foundation Trust; 11 2gether NHS Foundation Trust; Justine Brennan-Tovey, Cumbria, Northumbria and Tees Valley NHS Foundation Trust; 12 Oxleas NHS Foundation Trust; 13 NHS Lothian; 14 North East London NHS Foundation Trust; 15 Greater Manchester Mental Health NHS Foundation Trust; 16 Nottinghamshire Healthcare NHS Foundation Trust; 17 Sheffield Health and Social Care NHS Foundation Trust; 18 Avon and Wiltshire Mental Health Partnership NHS Trust; 19 Hywel Dda University Health Board; 20 Cheshire and Wirral Partnership NHS Foundation Trust; 21 University of Leeds; 22 Barnet, Enfield and Haringey Mental Health Trust and University College London

## Abstract

**Aims:**

The number of people over the age of 65 attending Emergency Departments (ED) in the United Kingdom (UK) is increasing. Those who attend with a mental health related problem may be referred to liaison psychiatry for assessment. Improving responsiveness and integration of liaison psychiatry in general hospital settings is a national priority. To do this psychiatry teams must be adequately resourced and organised. However, it is unknown how trends in the number of referrals of older people to liaison psychiatry teams by EDs are changing, making this difficult.

**Method:**

We performed a national multi-centre retrospective service evaluation, analysing existing psychiatry referral data from EDs of people over 65. Sites were selected from a convenience sample of older peoples liaison psychiatry departments. Departments from all regions of the UK were invited to participate via the RCPsych liaison and older peoples faculty email distribution lists. From departments who returned data, we combined the date and described trends in the number and rate of referrals over a 7 year period.

**Result:**

Referral data from up to 28 EDs across England and Scotland over a 7 year period were analysed (n = 18828 referrals). There is a general trend towards increasing numbers of older people referred to liaison psychiatry year on year. Rates rose year on year from 1.4 referrals per 1000 ED attenders (>65 years) in 2011 to 4.5 in 2019 . There is inter and intra site variability in referral numbers per 1000 ED attendances between different departments, ranging from 0.1 - 24.3.

**Conclusion:**

To plan an effective healthcare system we need to understand the population it serves, and have appropriate structures and processes within it. The overarching message of this study is clear; older peoples mental health emergencies presenting in ED are common and appear to be increasingly so. Without appropriate investment either in EDs or community mental health services, this is unlikely to improve.

The data also suggest very variable inter-departmental referral rates. It is not possible to establish why rates from one department to another are so different, or whether outcomes for the population they serve are better or worse. The data does however highlight the importance of asking further questions about why the departments are different, and what impact that has on the patients they serve.

